# Nursing Behaviors which Facilitate the Grief Work of Parents with Premature Infants in Neonatal Intensive Care Unit: A Comparison of Mothers and Fathers

**DOI:** 10.5812/nms.10369

**Published:** 2013-06-27

**Authors:** Elaheh Rahiminia

**Affiliations:** 1 School of Nursing and Midwifery, Tabriz University of Medical Sciences, Tabriz, IR Iran

**Keywords:** Grief, Parents, Premature birth, Nurses, Intensive Care Units, Neonatal

## Abstract

**Background:**

The birth of a child is an event to be remembered. However, a premature birth may shock the parents and cause their grief. Understanding differences between mothers and fathers can help nurses in providing nursing supports.

**Objectives:**

This study was performed with the aim of comparing nursing behaviors which facilitate grief work for parents of premature infants hospitalized in the NICU from perspectives of mothers and fathers.

**Patients and Methods:**

This comparative descriptive design was conducted among 40 pairs of mothers and fathers selected by convenience sampling method. The study was performed in 2011 using the Fordham Scale (1989). Data were analyzed with "wilcoxon ranks test" by using SPSS software version 13.

**Results:**

The mean scores of nursing behaviors which facilitate grief work were 2.81 ± 0.16 and 2.82 ± 0.29 in the perspectives of mothers and fathers, respectively. The Wilcoxon test did not show any statistically significant difference between mothers and fathers (P = 0.55).

**Conclusions:**

Despite expectations, provided nursing behaviors in mothers and fathers showed no difference in this study. Therefore, nursing policymakers and directors should take measures in order to provide appropriate services to the parents.

## 1. Background 

During the course of a pregnancy, families anticipate the birth of a child whose face resembles theirs, whose abilities will develop, and whose interests they can participate in. The birth of a premature neonate might thus be a crisis for every family (1). Premature neonate is a baby born before the gestational age of 37 weeks (2, 3). After the birth and admittance of the premature neonate to Neonatal Intensive Care Unit (NICU), grief work occurs in parents (4). Grief work is the emotional reactions to a significant loss which has psychological, cognitive, physiological, and behavioral manifestations (5-7). Grief physical responses includes weakness, anorexia, fatigue, insomnia, tightness in the chest, shortness of breath and psychological reactions such as overwhelming, sadness, guilt, loneliness, hopelessness, and anger (7-9). In Benfiled's (10) and valizadeh's (11) study, parents reported grief reactions such as feeling of sadness, anger, difficulty in sleeping and appetite, preoccupation thinking, irritability and guilt. In other words, unexpected and rapid events alter the roles and relationships of the parents with premature neonates and force them encounter with complicated situations (8, 12). Long-term hospitalization of premature neonates results in separation of infant and parents and interferes in the attachment process (13). Miles (14) categorized the nursing support for the parents of premature neonates in NICU into four fields of informative support, emotional support, self esteem, and qualitative care. Also, Fordham (15) categorized the nursing behaviors which facilitate the grief work for the parents of premature neonates in NICU into five dimensions of providing an environment that promotes personal development, supporting physically or psychologically, guiding, teaching, and acting. In order to promote the quality of the provided care, it is necessary to identify nursing supports used by nurses. The medical and nursing staff needs to recognize the special needs of parents with a premature infant in order to provide appropriate support. Nonetheless, few studies have been conducted from parents point of view regarding to this (15) and unfortunately, none has been conducted to the date in Iran.

## 2. Objectives

This study was performed with the aim of comparing nursing behaviors which facilitate grief work for parents of premature infants hospitalized in the NICU from perspectives of mothers and fathers. Our Hypothesis was that ‘The provided nursing behaviors to parents with premature infants hospitalized in the NICU are the equal for mothers and fathers’.

## 3. Materials and Methods

In a comparative descriptive study, parents of premature infants hospitalized in NICU of three medical centers in Tabriz, Iran, were investigated in 2011. The sample size was estimated after a pilot study with 10 pairs of the parents of premature infants hospitalized in the NICU by using two mean comparison formula with type I error α < 0.05 and power 0.9. Finally, 80 parents (40 mothers and 40 fathers) were selected by convenience sampling method during 4 months. The inclusion criteria were parents without apparent congenital malformations in their newborn, neonate between the fifth to twentieth days of hospitalization in the NICU, and the following characteristics in both of the parents: age over 18 years, no history of mental illness or being treated with psychotropic drugs, lack of prenatal consulting and observations from the NICU, no previous history of infant hospitalization in the NICU.

The data was collected using a questionnaire with two sections; part one was consistent of demographic questions and the second part was based on the 53 items related to the Fordham Scale (1989) of nursing behaviors which facilitate the grief work of parents with premature neonates in NICU (15), but was modified to 46 items for increasing the content validity based on the socio-cultural context of Iran. The questionnaire was based on a 4-point Likert scale which was categorized from 0 (Completely agree) to 4 (Completely disagree). The tool was classified in five dimensions: “Providing an environment that promotes personal development” (8 items), “Supporting another physically or psychologically” (13 items), “Guiding another” (6 items), “Teaching another” (9 items), and “Acting for or doing for” (10 items). The English original version of the Fordham Scale was translated to Persian by a forward–backward translation method. The content validity of the questionnaire was determined by nine of the Tabriz school of midwifery and nursing staff and two psychiatrics. After introducing the amendments, this version of the instrument was pilot tested with 10 pair of parent which yielded a Cronbach’s alpha of 0.79 for mothers, and 0.90 for fathers. The data collection was performed after having spent time caring for their infant at the NICU and in privacy and calm environment; Parents were interviewed through the questionnaire for approximately one hour by the researcher and well-trained assistant researcher.

This study was adopted by the Research Department of Tabriz University of Medical Sciences (Project Code No. 901). It was approved by the University Ethics Committee. Informed consent was obtained from all mothers and fathers (in order to participate in the study). Data collection was marked by an effort to protect participants from suffering and harm. The name of participants was not mentioned and principle of keeping secrets was observed. The confidentiality of personal information and maintaining the right to withdraw at any stage of research was fulfilled.

Data was analyzed with descriptive statistics (frequency distribution, percentage, mean, and standard deviation). Non-parametric Wilcoxon test is used for comparing the qualitative and ordinal variable of nursing behaviors in mothers and fathers who are two depended groups. SPSS software version 13 was used and P < 0.05 was considered significant.

## 4. Results

The mean maternal age was 26.3 ± 6.5 years (ranged 18 to 42). The maternal education level was primary, secondary, diploma and university in 8 (20%), 11 (27.5%), 11 (27.5%) and in 10 (25%) subjects, respectively. Sixty percent of the mothers had a cesarean delivery. Three (7.5%) mothers stayed out of the NICU but rest of them (37 out of 40 mothers) were hospitalized while the infant was transferred to NICU. The mean age of fathers was 30.8 ± 6.6 years (ranged 21 to 47). The fathers education level was primary in 6 (15%), secondary in 12 (30%), diploma in 8 (20%) and university in 14 (35%) subjects.

26 (68.4%) of the premature infants were the first child of the family. There were 19 (45.5%) male infants, 17 (42.5%) females and 4 (10%) twins. The mean gestational age of 40 infants was 30.2 ± 5.3 weeks (ranged 24 to 36). The mean birth weight was 2.97 ± 0.84 kg. Out of 40 premature infants, 38 (95%) were hospitalized with respiratory problems and 2 (5%) had a heart problem.

Table 1 Shows the Mean and Standard deviation of all dimensions of nursing behaviors which facilitate grief work.

**Table 1.** Dimensions of Nursing Behaviors Which Facilitate Grief Work of Parents with Premature Neonates in the NICU

Dimensions, (Mean ± SD)^ b^	Mothers	CI^a^, 95%	Father	CI, 95%
**Providing an environment that promotes personal development**	2.32 ± 0.30	2.22 - 2.42	2.47 ± 0.31	2.37 - 2.58
**Supporting another physically or psychologically**	2.96 ± 0.29	2.87 - 3.05	2.73 ± 0.45	2.58 -2.87
**Guiding another**	2.26 ± 0.36	2.14 - 2.38	2.39 ± 0.48	2.24 - 2.55
**Teaching another**	3.45 ± 0.20	3.38 - 3.51	3.50 ± 0.25	3.42 - 3.58
**Acting for or doing for**	2.73 ± 0.24	2.65 - 2.81	2.79 ± 0.35	2.68- 2.91
**Total**	2.81 ± 0.16	2.76 - 2.87	2.82 ± 0.29	2.73-2.92

^a^ Abbreviation: CI, confidence interval

^b^ The fewer score shows the better function

According to the Table 1, the highest mean score nursing behaviors from the perspective of the parents were "guiding another" and "providing an environment that promotes personal development". Mothers considered the dimensions of "teaching another" and "Supporting another physically or psychologically" as the least nursing behavior while fathers reported the dimensions of "teaching another" and "Acting for or doing for" as one of the least behaviors.

Also, Wilcoxon test showed no statistically significant difference between fathers and mothers

(P = 0.55, z = -0.59).

## 5. Discussion

The results of this study showed that both mothers and fathers of premature infants hospitalized in the NICU received the same nursing behaviors. Also in this study, hypothesis testing showed that there was no significant difference between the provided nursing behaviors for mothers and fathers. In our study the least recurrent nursing behavior from the perspective of parents was "teaching another". This finding is in accordance with Fordham’ study results (15). Parents would like to receive correct information about their hospitalized infants. They also need explanations, information shared and teaching related to their child's condition (16-19). Talking with parents, giving information, encouraging them to express their emotions, participating in caring activities, and referring them to support groups are some of the nursing interventions to help the parents of premature neonates (20).

Although mothers are in most contact with nurses because of their care role than fathers, nonetheless, the provided nursing behaviors in mothers and fathers showed no difference in this study. Therefore, it could mean neonatal nurses had less attention to parents even for mothers.

In lee's study (21) mothers of medically fragile infants introduced the fathers as a major supporter for themselves. Also in our study, after the dimension of "teaching another", mothers reported that "supporting another physically or psychologically" is one of the least applied behaviors whiles fathers reported "Acting for or doing for "as one of the least recurrent behaviors. It shows that mothers need more support than fathers. It is recommended that, nurses should be having their highest attention level for parents' support especially mothers. High quality nursing care for a neonate needs to be accompanied by emotional support, attention, encouragement and respect for the parents (22). According to Higman (23), nurses also confirmed that the parents need emotional support. Hutti (24) stated "Each parent and family must be assessed individually and appropriate interventions should beoffered from an evidence-based repertoire".

This study showed that "teaching", "supporting physically or psychologically" and "Acting for or doing for" were of the least applied behaviors. Therefore, continuing educational programs for nurses should be designed. Overally the ability of parents of premature neonates in coping with grief and the support offered by nurses for families (family-centered care) are of utmost importance. Our results may not be generalized to other parents or medical centers because this study was performed through convenient sampling, only among some parents of premature infants in Tabriz, Iran. Parents might have avoided telling the truth during interviews because of their fear of nurses not giving their neonates high quality care. This confounding error was partially controlled by gaining the parents' trust. Furthermore the effects of variables such as education, gender, social support, prior grief experiences, or personality factors on the perception of what nursing behaviors facilitate the grief work of parents with a premature infant in a neonatal intensive care unit need to be explored as these have not been resolved by the present study.

Results of study can help neonatal nurses and nursing directors to design supporting programs for nursing care in accordance with the needs of parents with premature infants. Information about the methods of assisting with the nursing behaviors which facilitate the grief work of parents with a premature infant in a neonatal intensive care unit should be developed to provide directions for nursing practice and curricula in schools of nursing. As there is no supportive program for parents with premature neonates in Iran, a qualitative study is recommended to help policymakers of the nursing profession compile a supportive program.
